# Specificity of Lipoxygenase Pathways Supports Species Delineation in the Marine Diatom Genus *Pseudo-nitzschia*

**DOI:** 10.1371/journal.pone.0073281

**Published:** 2013-08-27

**Authors:** Nadia Lamari, Maria Valeria Ruggiero, Giuliana d’Ippolito, Wiebe H. C. F. Kooistra, Angelo Fontana, Marina Montresor

**Affiliations:** 1 Istituto di Chimica Biomolecolare, Consiglio Nazionale delle Ricerche, Pozzuoli, Italy; 2 Laboratory of Ecology and Evolution of Plankton, Stazione Zoologica Anton Dohrn, Napoli, Italy; University of New South Wales, Australia

## Abstract

Oxylipins are low-molecular weight secondary metabolites derived from the incorporation of oxygen into the carbon chains of polyunsaturated fatty acids (PUFAs). Oxylipins are produced in many prokaryotic and eukaryotic lineages where they are involved in a broad spectrum of actions spanning from stress and defense responses, regulation of growth and development, signaling, and innate immunity. We explored the diversity in oxylipin patterns in the marine planktonic diatom *Pseudo-nitzschia*. This genus includes several species only distinguishable with the aid of molecular markers. Oxylipin profiles of cultured strains were obtained by reverse phase column on a liquid chromatograph equipped with UV photodiode detector and q-ToF mass spectrometer. Lipoxygenase compounds were mapped on phylogenies of the genus *Pseudo-nitzschia* inferred from the nuclear encoded hyper-variable region of the LSU rDNA and the plastid encoded *rbc*L. Results showed that the genus *Pseudo-nitzschia* exhibits a rich and varied lipoxygenase metabolism of eicosapentaenoic acid (EPA), with a high level of specificity for oxylipin markers that generally corroborated the genotypic delineation, even among genetically closely related cryptic species. These results suggest that oxylipin profiles constitute additional identification tools for *Pseudo-nitzschia* species providing a functional support to species delineation obtained with molecular markers and morphological traits. The exploration of the diversity, patterns and plasticity of oxylipin production across diatom species and genera will also provide insights on the ecological functions of these secondary metabolites and on the selective pressures driving their diversification.

## Introduction

Oxylipins are low-molecular-weight cyclic or acyclic products derived from the incorporation of oxygen into the carbon chains of polyunsaturated fatty acids (PUFAs), mediated by lipoxygenase (LOX) enzymes. These secondary compounds occur in many photosynthetic organisms including plants, algae and cyanobacteria (e.g., [1]), as well as in animals [2], [3] [4], [5], [6]) and fungi [7]. In higher plants, these molecules have evolved as primary defense in responses to physical damage by animals or insects, stress and attack by pathogens, and as regulators of growth and development [8], [9] [10], [11], [12], [13], [14]). In algae, LOX products have been reported in brown and red algae ([15], [16] [17]) and seem to be intermediates for innate immunity [18], [12]. Oxylipins have been reported also in diatoms [19], [20], [21]. These compounds are suggested to impair the reproductive biology of copepods, the principal plankton grazers, and to mediate intracellular communication (e.g. [22], [23], [24], [25], [26], [27], [28]). These studies also showed a high level of species-specific variability of diatom oxylipins that might indicate genetic [29] and physiological control of their biosynthesis [30], [31], [32], [26].

The planktonic pennate diatom genus *Pseudo-nitzschia* is encountered in marine environments all over the world and includes ca. 30 taxonomically described species [33]. *Pseudo-nitzschia* cells are needle-shaped and form stepped-chains by aligning tip-to-tip. Species identification is based on cell shape and width, cell overlapping pattern in the chain, and ultrastructural features of the siliceous frustule [34]. Several groups of morphologically highly similar or even identical species occur, which can only be told apart using rapidly evolving DNA-markers such as the internal transcribed spacer region (ITS) and the hyper-variable domain (D1-D3) of the large subunit (LSU) in the nuclear ribosomal RNA cistron [33]. For instance, the morpho-species *P* *. delicatissima* consists of at least four genetically distinct species: *P* *. delicatissima* ‘*sensu stricto*’, *P. arenysensis* *,* *P* *. decipiens* , and *P* *. dolorosa* [35], [36] [37]. Many of these species occur in sympatry in our study area, the Gulf of Naples in the Mediterranean Sea [37].

Recently we described a novel protocol for rapid characterization of oxylipins in lipid extracts of diatoms by reverse phase liquid chromatography and tandem quadrupole-ToF mass spectrometry (qToF LC-MS/MS) [21]. In addition to traditional metabolite profiling, the method permits to deduce the positional specificity of lipoxygenation by MS/MS fragmentation of hydroxy-epoxy PUFA derivatives, generically named epoxy-alcohols, which are among the major classes of oxylipins so far reported in diatoms. Thus, the qToF LC-MS/MS analysis provides a powerful tool to investigate distribution of LOX activities among different diatom samples. The aims of the present study were to assess: i) the spectrum of oxylipins produced by different *Pseudo-nitzschia* species, i.e., if one or more compounds are produced per monoclonal strain; ii) if oxylipin composition changes among strains within the same genetically defined species; and iii) if a match exists between phylogenetic relationship and LOX profiles, i.e., if closely related species produce the same compounds and more distantly related species different ones, and in addition, if phylogenetically closely related *Pseudo-nitzschia* species produce more similar oxylipins than more distantly related species. Strains were genetically identified based on the hyper-variable region (ca. 700 base pairs) at the 5’-end of their nuclear-encoded LSU rDNA and on the plastid-encoded large subunit of their ribulose-1,5-bisphosphate carboxylase/oxygenase (*rbc*L). These markers have been shown to differ among biologically distinct species in *Pseudo-nitzschia* [31], [38].

## Materials and Methods

### Diatom cultures

*Pseudo-nitzschia* strains were isolated at the Long Term Ecological Research Station MareChiara in the Gulf of Naples (Tyrrhenian Sea, Mediterranean Sea) (Table S1). *Pseudo-nitzschia multiseries* strain NWFSC 316 was isolated in Clam Bay, Puget Sound, USA (Table S1). Clonal cultures were established by isolating single cells or short chains from phytoplankton net samples collected in the surface layer of the water column. Cultures were grown in sterile filtered oligotrophic seawater amended with f/2 nutrients [39] and maintained at a temperature of 20°C, a 14: 10h light: dark cycle, and with a photon flux of 100 μmol photons m^-2^ s^-1^.

### Molecular characterization

A 25 ml subsample was collected from each culture in exponential growth phase and genomic DNA was extracted following [40]. DNA was amplified by PCR using the primers D1R and D3Ca [41], [42] for the hypervariable domains D1 and D3 of the LSU rDNA and two degenerated primers, rbcL1 and rbcL7 [37] for the *rbc*L. PCR conditions and sequence analyses were carried out as reported in [37]. The accession number of the sequences produced within this investigation is reported in Table S1. Additional LSU rDNA and *rbc*L sequences of other *Pseudo-nitzschia* species were downloaded from GenBank, aligned by eyeball and used to build Maximum Likelihood phylogenies. Strain codes and GenBank accession numbers are reported in Table S1. Maximum-likelihood trees were inferred based on the substitution models selected through the Akaike information criterion option implemented in jModeltest [43] using MEGA5 [44], with 100 bootstrap replicates. Best fit models were GTR + I + G (I= 0.59; γ = 0.2266) for *rbc*L and GTR + I + G (I= 0.69; γ = 0.2180) for LSU.

### Chemical analyses

To obtain the biomass required for chemical analyses, cultures were grown in Erlenmeyer flasks filled with 1 L f/2 medium at the conditions illustrated above. Cell concentration was monitored over time by collecting a 2-ml subsample that was used for estimating cell numbers with a Sedgewick-Rafter counting chamber utilizing a Zeiss Axiophot microscope (Karl Zeiss, Oberkochen, Germany). Cells were harvested at the stationary phase by centrifugation at 1200 g for 10 min at 5°C. The cell pellets were collected in 50 ml Falcon tubes, immediately frozen in liquid nitrogen, and kept at -80°C until chemical analysis was performed. Each strain was treated as an independent entry in the chemical analysis, irrespective of its taxonomic assignment; some strains have been grown and analysed on different occasions to test for constancy of their oxylipin pattern (Table S1). When possible, replicates of the chemical analyses were also carried out.

Cell pellets were dissolved in distilled water (1 ml·g^-1^ of wet pellet) and sonicated for 30 sec at 4°C prior to extraction with methanol (equal volume with water). The internal standard, 16-hydroxyhexadecanoic acid (15 μg·g^-1^ of pellet) was added before the extraction, and the resulting suspension was centrifuged at 2000 g for 5 min at 5°C. The organic extracts were dried under reduced pressure with a rotary evaporator (Buchi, Rotavapor R-200) and then methylated with ethereal diazomethane in diethyl ether (0.4 ml per 10 mg extract) for 1 hr at room temperature. After removing the organic solvent under nitrogen stream, the raw extract was used for analysis of non-volatile oxylipins by a Waters Alliance liquid chromatography equipped with UV photodiode detector (PDA) and Micromass *micro*q-ToF (qToF) mass spectrometer (Waters, Milford, MA, USA) as described in [21]. Identification of individual products was supported by matching of primary analytical indicators (PAIs), (retention time, UV spectrum, molecular weight and mass/mass fragmentation) with those experimentally determined (standards) or calculated [21]. Determination of lipoxygenase positional specificity is based on mass/mass analysis of epoxy-alcohols. Oxylipin levels were established by normalizing the component peak area by the peak area of the internal standard (16-hidroxyhexadecaoic acid methyl ester).

## Results

### Molecular phylogeny

**Figure 1:**
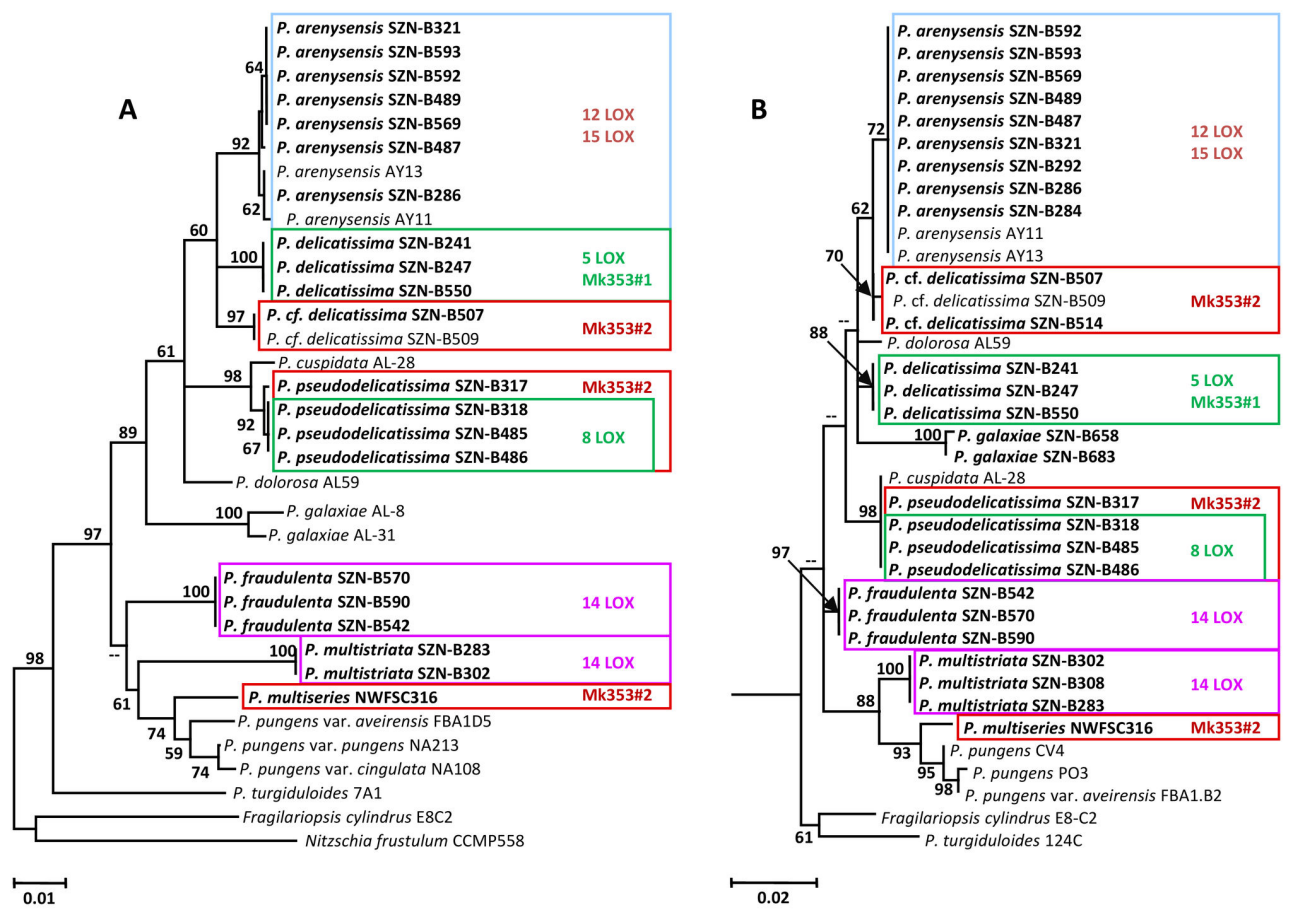
Molecular phylogeny of *Pseudo-nitzschia* species. Maximum Likelihood tree constructed with *rbc*L (A) and LSU (B) sequences. The strains in bold have been analysed for oxylipin production. The position specificity of the LOX activity detected for the different strains and the still unidentified compounds are mapped with different colours on the trees.

Maximum likelihood inference of the *rbc*L sequences provided a well-resolved phylogeny (Figure 1A). *Pseudo-nitszchia turgiduloides* was recovered at a basal position and the remainder *Pseudo-nitzschia* species grouped into three main clades. One of these included all *P* *. fraudulenta* strains, the second one contained a clade with strains of *P* *. multistriata* as sister to a clade with strains of *P*. *multiseries* and *P* *. pungens* , and a third one contained a clade with stains of *P* *. galaxiae* as sister to a polytomy comprising species specific clades with strains of *P* *. pseudodelicatissima* and all species of the *P* *. delicatissima* complex ( *P* *. delicatissima* , *P. dolorosa* *,* *P* *. arenysensis* ). Within this third clade, strains SZN-B507 and SZN-B509 grouped together. The two strains showed morphological and ultrastructural characters very similar to those of *P* *. delicatissima* (D. Sarno, personal communication) and their sequences did not match with any sequence deposited in GenBank. We thus considered these strains as a new species, and provisionally designated it as P. cf. *delicatissima* new genotype (D. Sarno, MV Ruggiero and collaborators, manuscript in preparation). The *rbc*L marker separated *P* *. pseudodelicatissima* from *P* *. cuspidata* . Intraspecific genetic differences were evident among strains of *P* *. arenysensis* and *P* *. pseudodelicatissima* in the form of single nucleotide differences. Remarkably large genetic diversity was instead evident within the *P* *. galaxiae* clade*.*

The LSU rDNA tree (Figure 1B) revealed the same terminal clades as the *rbc*L tree, with the following exceptions; the marker did not discriminate *P* *. pseudodelicatissima* from *P* *. cuspidata* and did not show differences amongst *P* *. arenysensis* strains. In addition, *Fragilariopsiscylindrus* grouped with *P* *. turgiduloides* , though with a low bootstrap support. Relationships among the terminal clades were less well resolved than in the *rbc*L tree as shown by low or lacking bootstrap support and by the recovery of polytomies. Nonetheless, the topologies between the two trees showed no conflicts. We did not obtain *rbc*L sequences for the two *P* *. galaxiae* strains analysed for oxylipin composition. However, we included in the phylogenetic analysis the *rbc*L sequences of two other *P* *. galaxiae* strains whose LSU sequences were identical to LSU sequences of the analysed strains (see Table S1). The *rbc*L sequences of those *P* *. galaxiae* strains showed a remarkably higher level of nucleotidic divergence with respect to LSU.

### Chemical analyses

**Figure 2:**
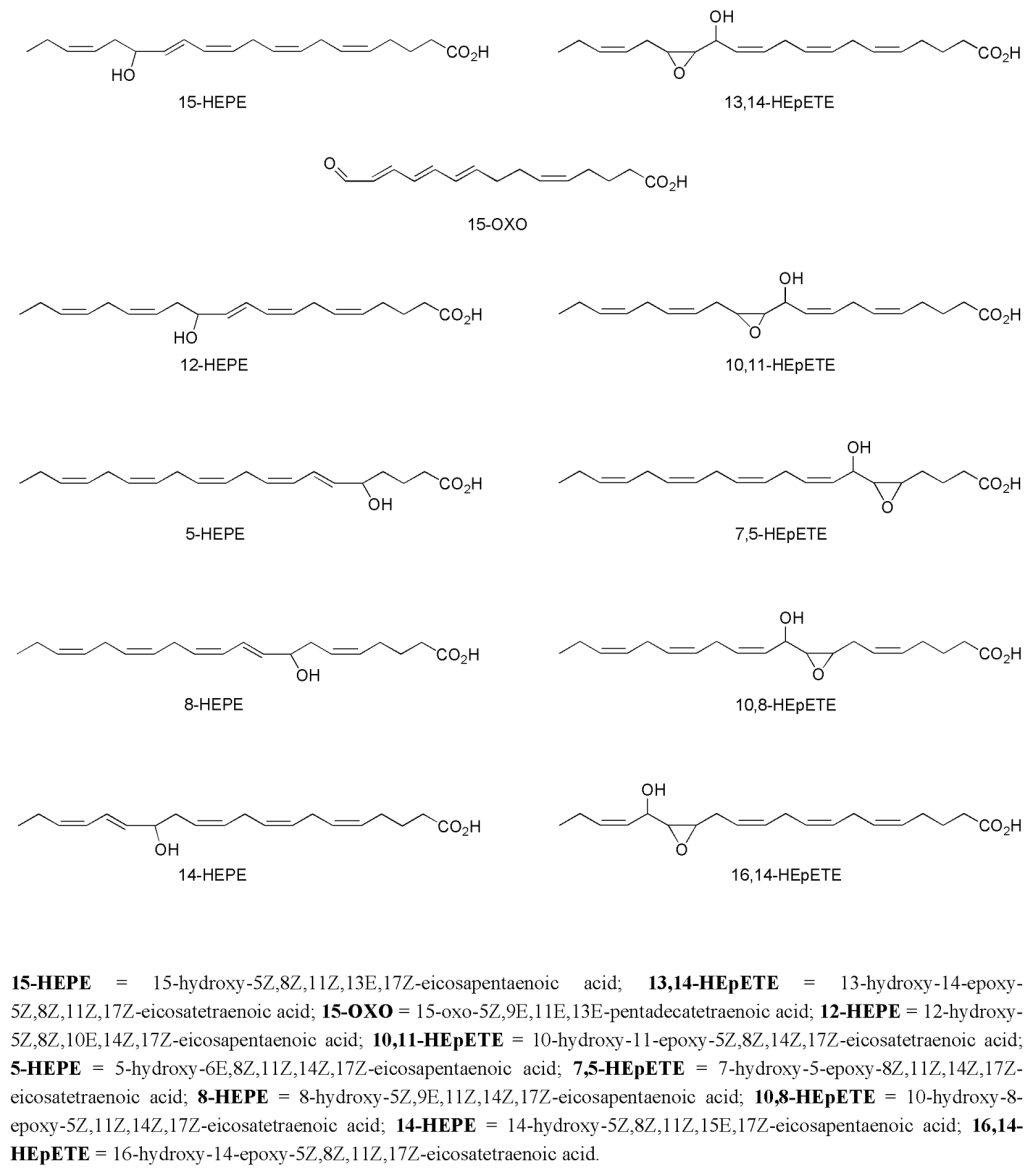
Major EPA-derived oxylipins characterized in *Pseudo-nitzschia* species during this study. Compounds have been identified on the basis of comparison of primary analytical indicators (retention time, UV spectrum, molecular weight and mass/mass fragmentation) with those experimentally determined or calculated in agreement with [21].

**Figure 3:**
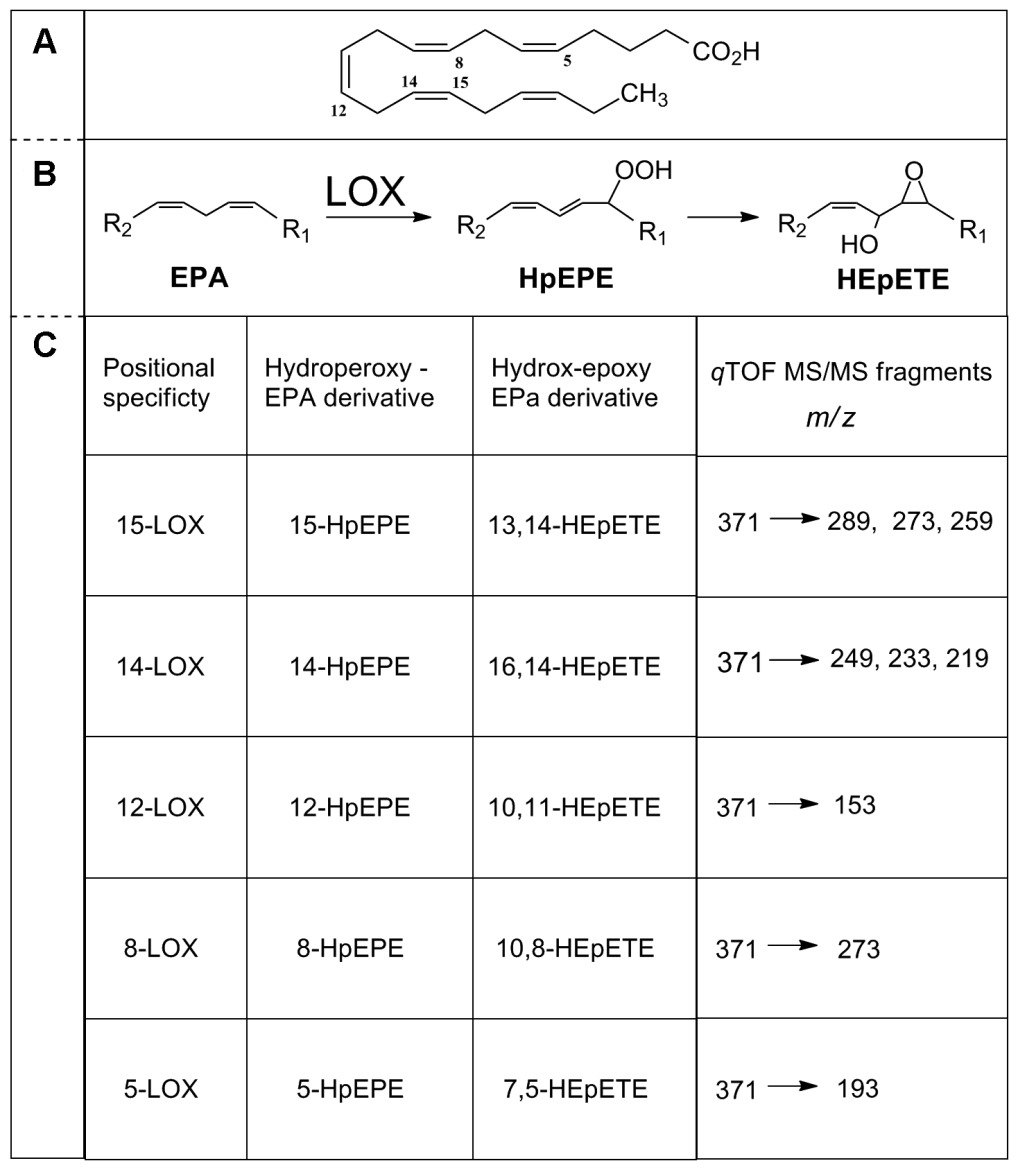
Lipoxygenase positional specificity in the genus *Pseudo-nitzschia*. Identification is inferred on MS/MS fragmentation of hydroxy-epoxy eicosatetraenoic acids (HEpETEs) derived from LOX-mediated metabolism of EPA. (**a**) EPA and position of LOX oxidation. (**b**) Biochemical mechanism leading to specific transformation of primary LOX product (hydroperoxy-eicosapentaenoic acid, HpEPE) to HEpETE. For simplicity, the polyunsaturated chain of EPA is represented by the 1,3-pentadiene moiety that undergoes to enzymatic oxidation. R_1_ and R_2_ are variable alkyl residues to complement the structure of EPA. (**c**) Diagnostic ions of different HEpETEs generated by MS/MS fragmentation.

**Figure 4:**
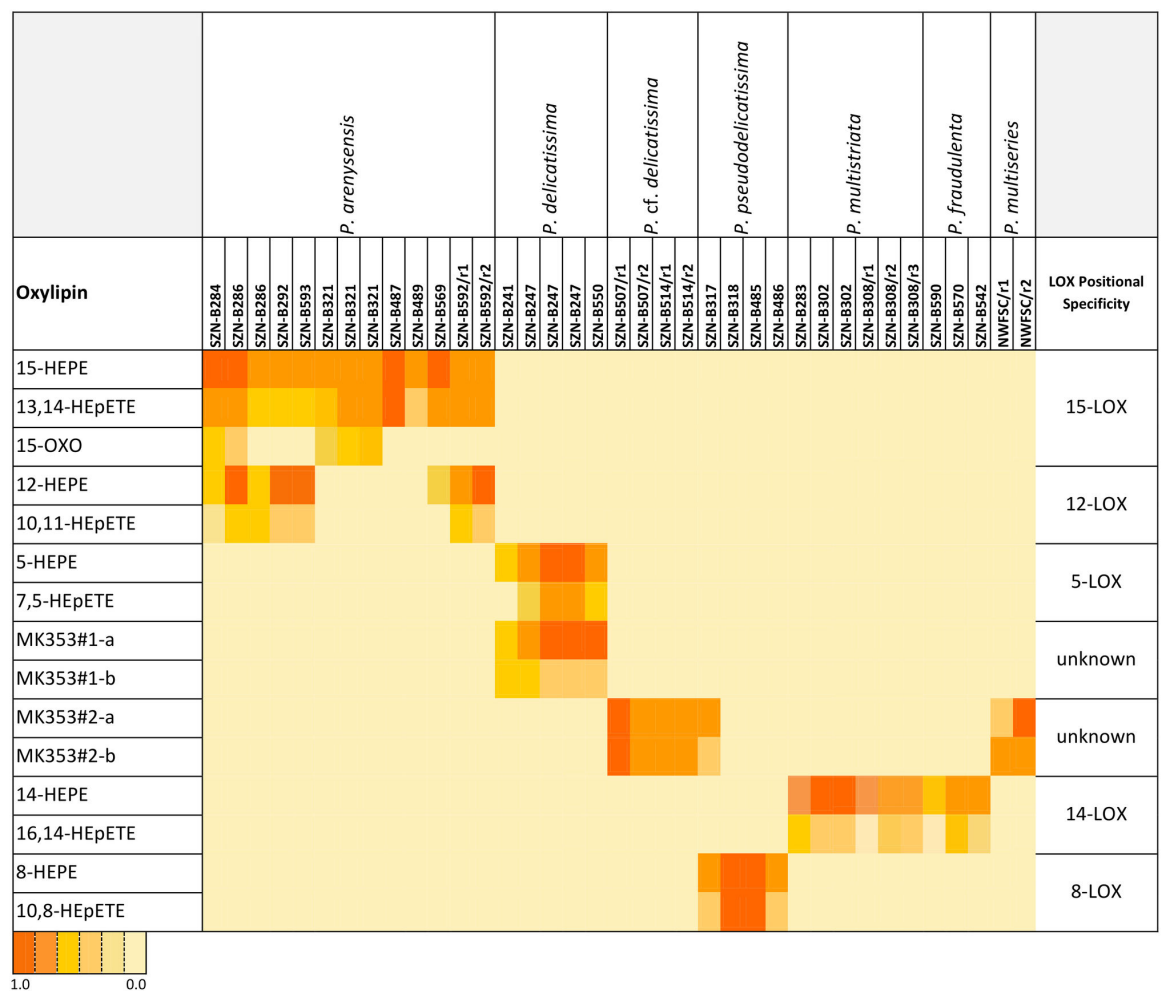
Heat map representation of oxylipin signals in the *Pseudo-nitzschia* strains. A semi-quantitative estimate of the individual oxylipins (rows) recorded in the analyzed strains (columns). Color scale represents the ratio between peak areas of individual oxylipins and internal standard (see Materials and Methods). Suffix ‘/r’ followed by number indicates biological replicates of the same strain. Chemical abbreviations are in agreement with Figure 2.

Of the eight *Pseudo-nitzschia* species tested (Table S1), only the two strains of *P* *. galaxiae* did not show unequivocal presence of oxylipins, although traces of molecules possibly derived from C18- and C20- polyunsaturated fatty acids, were detected. LC-MS/MS profiles were generally consistent amongst different strains (Table S1) belonging to the same species as well as between replicates of chemical analyses carried out on the same samples, whereas they differed significantly among species. Figure 2 reports the oxylipins identified in the diatom strains during this study. All these compounds derive from eicosapentaenoic acid (EPA) and can be grouped in two major families containing hydroxy-eicosapentaenoic acids (HEPEs) and hydroxy-epoxy eicosatetraenoic acids (HEpETEs). Only a few samples of *P* *. arenysensis* also revealed presence of 15-oxo-5Z,9*E*,11*E*,13*E*-pentadecatetraenoic acid (15-OXO) [26]. Thus, the diversity of oxylipin profile in the *Pseudo-nitzschia* samples stemmed only from changes in lipoxygenation site of EPA. This feature is associated to positional specificity of LOX enzymes, which was inferred by analysis of the fragmentation pattern of HEpETEs in MS/MS analysis (Figure 3) [21]. According to these results, marked variations were detected among the *Pseudo-nitzschia* species (Figures 1, 4). Within the *P* *. delicatissima* complex, all *P* *. arenysensis* strains clustered for the presence of 15- and 12-LOX-derived compounds, namely 15-hydroxy-5*Z*,8*Z*,11*Z*,13*E*,17*Z*-eicosapentaenoic acid (15-HEPE), 13-hydroxy-14-epoxy-5*Z*,8*Z*,11*Z*,17*Z*-eicosatetraenoic acid (13,14-HEpETE), 15-OXO, 12-hydroxy-5*Z*,8*Z*,10*E*,14*Z*,17*Z*-eicosapentaenoic acid (12-HEPE) and 10-hydroxy-11-epoxy-5*Z*,8*Z*,14*Z*,17*Z*-eicosatetraenoic acid (10,11-HEpETE). These products generally co-occurred in the species even if only 15-LOX activity was detected in strains SZN-B593, SZN-B321 and SZN-B487, as well as only oxylipins derived from 12-LOX metabolism were found in strain SZN-B489. On the contrary, the three samples of *P* *. delicatissima* exhibited metabolites derived from the 5-LOX pathway: 5-hydroxy-6*E*,8*Z*,11*Z*,14*Z*,17*Z*-eicosapentaenoic acid (5-HEPE) and 7-hydroxy-5-epoxy-8*Z*,11*Z*,14*Z*,17*Z*-eicosatetraenoic acid (7,5-HEpETE). This species also produced a pair of isomeric oxylipins (R_t_= 24.2 and 21.6 min) with MS molecular ion at 353 (M+Na^+^). Both compounds showed UV λ_max_ at 248 nm in agreement with a conjugated keto C-20 oxylipin but, since the amount of the sample did not allow completion of the structure assignment, they have been designed as undefined chemical markers of group 1 (Mk353#1). Two other oxygenated fatty acids (R_t_= 21.2 and 18.4 min) with molecular mass of *m/z* 353 characterized the MS profiles of *P. pseudodelicatissima* strains. In analogy with Mk353#1, these data suggested the presence of another pair of isomeric keto derivatives of EPA but their structures remain not fully determined and these compounds are thus reported as undefined chemical markers of group 2 (Mk353#2). This last pair of products was also detected in the new genotype of P. cf. *delicatissima* and in the distantly related *P* *. multiseries* .

*P* *. multistriata* and *P* *. fraudulenta* , which form a grade in Figure 1, contained 14-LOX activity that was inferred from MS/MS fragmentation of 16-hydroxy-14-epoxy-5*Z*,8*Z*,11*Z*,17*Z*-eicosatetraenoic acid (16,14-HEpETE). With the exception of strain SZN-B317, that differed from others strains in *rbc*L sequence, the MS data of *P* *. pseudodelicatissima* were fully consistent with the occurrence of 8-LOX products, namely 8-hydroxy-5*Z*,9*E*,11*Z*,14*Z*,17*Z*-eicosapentaenoic acid (8-HEPE) and 10-hydroxy-8-epoxy-5*Z*,11*Z*,14*Z*,17*Z*-eicosatetraenoic acid (10,8-HEpETE).

## Discussion

### Species specificity of LOX activity

The results of the present study show that the genus *Pseudo-nitzschia* exhibits a rich and varied LOX metabolism of eicosapentaenoic acid (EPA). As shown in Figure 4 that reports occurrence and relative abundance of individual oxylipin in the diatom samples undergone to qToF LC-MS/MS analysis, LOX positional specificity is suggestive of at least four chemotypes corresponding to 15- and 12-LOX in *P* *. arenysensis* , 5-LOX in *P* *. delicatissima* , 8-LOX in *P* *. pseudodelicatissima* and 14-LOX in *P* *. fraudulenta* and *P* *. multistriata* . Other possible chemotypes are associated to the presence of the unidentified compounds of group 1 and 2 (Mk353#1 and Mk353#2).

The species-specificity of these chemotypes was generally in good agreement with the molecular phylogenies based on *rbcl* and LSU, though not in all cases. For instance, the strains belonging to the distinct species *P* *. fraudulenta* and *P* *. multistriata* form a grade in the *rbc*L tree. Yet, they share 14-LOX, rendering the production of this compound probably a shared ancestral feature. Moreover, the fact that compound Mk353#2 is shared amongst different, not particularly closely related, species, suggests that the ability to produce this compound has been acquired at least twice, and possibly three times independently, or it has been acquired once and, subsequently, has gone lost in several species. In addition, each species produces just one or a few oxylipins. A notable exception is *P* *. galaxiae* , for which only traces of undefined oxygenated derivatives of fatty acids were detected. Our results support those of previous studies [26,45] that *Pseudo-nitzschia* lacks a distinctive trait of other planktonic diatoms, namely the LOX oxidation of C16 polyunsaturated fatty acids [46], [47], [48], [49], [45], [25].

Two LOX pathways appear to co-exist in *P* *. arenysensis* . Grouping of strains according to the minor *rbc*L differences amongst them did not corroborate a grouping according to 12- and 15-LOX products. Since LOX activity in diatoms seems to be modulated along the growth phase when maintained in culture [26], differences in LOX patterns could be due to physiological differences among the strains at the time of harvesting. Alternatively, this strain differentiation could be the result of genetic variation of LOX enzyme. In fact, single mutations of the peptide chain has been reported to induce modification of the fatty acid binding pocket, converting a 15-lipoxygenating enzyme into a 12-lipoxygenating enzyme and *vice versa* [50] [51] [52] (for a general discussion see [53]). The observation that some strains possess both compounds and others just one of these could then be explained by the fact that diatoms are diploid and that some of the strains are 12-LOX or 15-LOX homozygous and others 12-LOX + 15-LOX heterozygous.

Also *P* *. pseudodelicatissima* strains show minor differences in the *rbc*L sequences. Notably, the strain SZN-B317 that lacks 8-HEPE and 10,8-HEpETE also differs in the *rbc*L signature from the three strains that possess 8-LOX activity. In addition to the products reported in Figure 2, some of the species show compounds that were not conclusively characterized, but correspond to two sets of undisclosed LOX products (MK353#1 and MK353#2) with identical molecular weight. These products characterize all species of the apical *rbc*L clade grouping *P. pseudodelicatissima* *, P*, cf. *delicatissima* - new genotype, and *P* *. delicatissima* , but appear to have gone lost in *P* *. arenysensis* . Interestingly, the compound MK353#2 also characterizes *P* *. multiseries* , which is - according to the *rbc*L tree - not the sister of the abovementioned species. Previous studies on *P* *. multiseries* have established the presence of C20-derived lipoxygenase products, namely bacillariolides, that can be structurally related to the uncharacterized molecules reported here ( [54] [55]).

### Oxylipins as identification markers of *Pseudo-nitzschia* species

Traditionally, identification and delineation of unicellular microalgal species is based on phenotypic features such as cell size and shape and fine ultrastructural characters of their cell wall. Nucleotide markers have increased the precision of this identification process and have uncovered numerous cases of several genetically distinct lineages existing within single phenotypically delineated species (e.g. [56]). Also in diatoms, morphological differentiation has not held equal pace with differentiation into genetically and biologically distinct species, as shown by the discovery of numerous cases of genetically and biologically distinct species that are either morphologically indistinguishable (cryptic species) or can be separated only by minor ultrastructural details of their siliceous frustule (pseudo-cryptic species) (e.g. [57], [37] [58], [38],).

Differences in lipoxygenase products among a series of strains within *Pseudo-nitzschia* corroborate their genotypic delineation, even among genetically closely related cryptic species within the *P* *. delicatissima* complex, suggesting that these products can aid identification of species more precisely than morphological characters. This is particularly relevant when the presence of different epoxy-alcohols allows the rational deduction of the enzymatic dioxygenation site of the EPA polyunsaturated chain and, consequently, the classification according to the positional specificity of the putative LOXs. The three species within the complex ( *P. arenysensis* *, P, delicatissima, P*, cf. *delicatissima* new genotype) are indistinguishable in light microscopy and present only minor morphometric differences in the density of the poroids on their siliceous frustule visible in EM ( [37], D Sarno, MV Ruggiero and co-authors, manuscript in preparation for *P*, cf. *delicatissima* new genotype). The fact that closely related species show different LOX positional specificity seems to confirm that genetic changes accumulate rapidly in the enzymes of this pathway in *Pseudo-nitzschia*.

Several studies tested the Biological Species Concept sensu [59] in diatoms by carrying out mating experiments between sympatric strains of the same species as defined with morphological and molecular characters (e.g. [60], [37], [61]). The approach has been deployed also to delineate several of the *Pseudo-nitzschia* species included in the present investigation [37]. The corroboration of oxylipin distribution, LOX positional specificity and species assignment of the strains used in the present study suggests that LC-MS/MS profiling constitutes a promising support for molecular identification of *Pseudo-nitzschia* species. However, more data, including analyses of strains from other geographic regions, need to be screened for their oxylipin profiles to test if these profiles are species specific.

Secondary metabolites have been used successfully as markers for species delimitation in fungi [62] [63] or in ciliates [64]. Chemotaxonomic approaches in fungi are based both on the use of one specific metabolite or on metabolite profiles that allow the identification of distinct chemotypes. At times, chemotypes matched with groups based on sequence data, at times not, giving support to the hypothesis that chemical profiling is useful for species recognition, but not for inferring phylogenies [63]. Oxylipin profiling and related MS/MS expansion can assist in the species identification of *Pseudo-nitzschia* strains, but they cannot be used as sole taxonomic identification tool, as is illustrated by the different LOX activities among the *P* *. arenysensis* strains and among the *P* *. pseudodelicatissima* strains as well as by the sharing of the same LOX profiles among different species. Clearly, no single marker constitutes the univocal criterion for species recognition, as markers may not accurately reflect species diversity. Different nucleotide markers can provide conflicting evidence about species boundaries [65]. Moreover, the very process of speciation implies an *interim* phase during which species delineation is, by definition, impossible. A way forward to strive for accurate species delineation - and to identify cases where that is impossible - is to deploy different and complementary types of identifiers. Examples of this approach provide promising results. Sympatric pseudo-cryptic species have been detected in the benthic estuarine diatom *Navicula phyllephyta* based on morphological and molecular investigations; these species showed different growth responses along a salinity gradient, thus suggesting that they occupy distinct ecological niches [66]. Also the planktonic diatom morpho-species *Chaetoceros* *socialis* conceals pseudo-cryptic genotypes with apparently different geographic ranges [67]. Qualitative and quantitative differences of the metabolomic profiles of the two genotypes suggest that they evolved distinct adaptive capabilities to the contrasting temperature characteristics of their environment [68].

The fact that *Pseudo-nitzschia* species produce one or two isomeric forms of oxylipins and not a broad spectrum of these compounds suggests the existence of specific metabolic pathway to produce them. The distinct oxylipin profiles among closely related species indicate that the profiles change rapidly and suggest that selective pressure drives the change. The nature of such a selective pressure depends on the roles of these compounds and on their specific targets [69]. Diatom oxylipins may function as species-specific mediators of bloom control [24], [26]. Species-specific chemical communication requires species-specific compounds and species-specific receptors. Hence, the receptors need to evolve in concert with the compounds. In case the oxylipins function as signaling tools amongst specimens within the same *Pseudo-nitzschia* species then different species producing the same compounds should not co-occur, or they should produce additional compounds that, together, function as species-specific communication. Moreover, the observation that strains belonging to the same species produce different LOX-profiles suggests that some compounds could serve for purposes other than intraspecific communication. In fact, in addition or alternatively, LOX pathways may play a key role in producing defence chemicals in the arms race against grazers [70]. Such a function favours rapid changes in the types of oxylipins produced to outwit the copepods’ detoxification systems. The ensuing arms race is then expected to result in differences in LOX products among closely related species, or even between geographical populations of the same species. If the compounds serve as grazer deterrent, then intraspecific differences and interspecific identity do not pose any problem.

## Supporting Information

Table S1 ***Pseudo-nitzschia* strains analysed for oxylipin production.** Species name and strain code, date in which the chemical characterization was carried out, LSU rDNA GenBank accession number, *rbc*L GenBank accession number. When LSU and *rbc*L sequences were identical to sequences already deposited in GenBank, the reference number of the deposited sequences is provided.
(DOCX)
