# Supplementary material for: Specificity of Lipoxygenase Pathways Supports Species Delineation in the Marine Diatom Genus Pseudo-nitzschia
Source: PLoS One. 2013 Aug 27;8(8):e73281. doi: 10.1371/journal.pone.0073281 (PMC3754938; doi:10.1371/journal.pone.0073281)
Supplement: Table S1 — Pseudo-nitzschia strains analysed for oxylipin production. Species name and strain code, date in which the chemical characterization was carried out, LSU rDNA GenBank accession number, rbcL GenBank accession number. When LSU and rbcL sequences were identical to sequences already deposited in GenBank, the reference number of the deposited sequences is provided. (DOCX) [file pone.0073281.s001.docx]

Table S1

| **Species names and strain codes** | **Date of analysis** | **LSU rDNA** | ***rbc*L** |
| --- | --- | --- | --- |
| *P. arenysensis* |  |  |  |
| SZN-B284 | Jan 2007 | as DQ813811 |  |
| SZN-B286 | Mar 2007 | as DQ813811 | as DQ813823 |
| SZN-B286 | Jun 2007 |  |  |
| SZN-B292 | Jan 2007 | as DQ813811 |  |
| SZN-B593 | Nov 2011 | as DQ813811 | as DQ813819 |
| SZN-B487 | Apr 2009 | as DQ813811 | KC801036 |
| SZN-B489 | Apr 2009 | as DQ813811 | as DQ813819 |
| SZN-B569 | Nov 2011 | as DQ813811 | as DQ813819 |
| SZN-B592 | Nov 2011 | as DQ813811 | as DQ813819 |
| SZN-B321* | Nov 2007 | as DQ813811 | as DQ813819 |
| *P. delicatissima* |  |  |  |
| SZN-B247 | Nov 2006 | as DQ813810 | as DQ813818 |
| SZN-B247 | Mar 2007 |  |  |
| SZN-B247 | Jun 2007 |  |  |
| SZN-B241 | Jan 2007 | as DQ813810 | as DQ813818 |
| SZN-B550 | Nov 2011 | as DQ813810 | as DQ813818 |
| *P.*cf. *delicatissima* new genotype |  |  |  |
| SZN-B507 | Aug 2010 | KC801041 | KC801037 |
| SZN-B514 | Jul 2010 | KC801043 |  |
| *P.pseudodelicatissima* |  |  |  |
| SZN-B317 | Apr 2007 | as DQ813808 | KC801039 |
| SZN-B318 | Apr 2007 | as DQ813808 | as DQ813817 |
| SZN-B485 | Apr 2009 | as DQ813808 | as DQ813817 |
| SZN-B486 | Apr 2009 | as DQ813808 | as DQ813817 |
| *P. galaxiae* |  |  |  |
| SZN-B658 | May 2011 | as EF506606 |  |
| SZN-B683 | May 2011 | as EF522113 |  |
| *P. multistriata* |  |  |  |
| SZN-B283 | Nov 2006 | asAF416753 | as EF423505 |
| SZN-B302 | Jan 2007 | as AF416753 | as EF423505 |
| SZN-B302 | Mar 2007 |  |  |
| SZN-B308 | Jun 2007 | as AF416753 |  |
| *P. fraudulenta* |  |  |  |
| SZN-B590 | Nov 2010 | as EF522111 | as EF520333 |
| SZN-B570 | Nov 2010 | as EF522111 | as EF520333 |
| SZN-B542 | Nov 2010 | as EF522111 | as EF520333 |
| *P. multiseries* |  |  |  |
| NWFSC-316 | Nov 2011 | as AF440772 | KC801040 |

(*)Strain analysed in [[26](#_ENREF_26)]. The species was at that time identified as *Pseudo-nitzschia delicatissima* genotype 1, as reported in [31]; this genotype was later described as *P. arenysensis* [[36](#_ENREF_36)].
